# Nx4 Modulated Resting-State Functional Connectivity Between Amygdala and Prefrontal Cortex in a Placebo-Controlled, Crossover Trial

**DOI:** 10.1089/brain.2021.0189

**Published:** 2022-11-10

**Authors:** Tara Chand, Sarah Alizadeh, Meng Li, Yan Fan, Hamidreza Jamalabadi, Lena Danyeli, Melanni Nanni-Zepeda, Luisa Herrmann, Johan Van der Meer, Johannes C. Vester, Myron Schultz, Britta Naschold, Martin Walter

**Affiliations:** ^1^ Department of Psychiatry and Psychotherapy, University of Tübingen, Tübingen, Germany.; ^2^ Department of Psychiatry and Psychotherapy, Jena University Hospital, Jena, Germany.; ^3^ Department Psychology and Neurosciences, Leibniz Research Centre for Working Environment and Human Factors at the TU Dortmund (IfADo), Dortmund, Germany.; ^4^ Department of Psychiatry and Psychotherapy, Philipps-Universität Marburg, Marburg, Germany.; ^5^ Department of Radiology and Nuclear Medicine, Amsterdam UMC, Amsterdam, The Netherlands.; ^6^ idv Data Analysis and Study Planning, Gauting, Germany.; ^7^ Heel GmbH, Baden-Baden, Germany.

**Keywords:** amygdala, brain networks, complementary therapies, fMRI, resting-state functional connectivity

## Abstract

**Background:**

The basic functional organization of the resting brain, assessed as resting-state functional connectivity (rsFC), can be affected by previous stress experience and it represents the basis on which subsequent stress experience develops. Notably, the rsFC between the amygdala and the cortical regions associated with emotion regulation and anxiety are affected during stress. The multicomponent drug Neurexan^®^ (Nx4) has previously demonstrated a reduction in amygdala activation in an emotional face matching task and it ameliorated stress-related symptoms. We, thus, investigated the effect of Nx4 on rsFC of the amygdala before stress induction compared with baseline in mildly to moderately stressed participants.

**Methods:**

In a randomized, placebo-controlled, double-blind, crossover trial 39 participants received a single dose of placebo or Nx4. Resting-state functional magnetic resonance imaging scans were performed pre-dose and 40 to 60 min post-dose, before any stress induction. First, highly connected functional hubs were identified by global functional connectivity density (gFCD) analysis. Second, by using a seed-based approach, rsFC maps of the left centromedial amygdala (CeMA) were created. The effect of Nx4 on both was evaluated.

**Results:**

The medial prefrontal cortex was identified as a relevant functional hub affected by Nx4 in an explorative whole brain gFCD analysis. Using the seed-based approach, we then demonstrated that Nx4 significantly enhanced the negative connectivity between the left CeMA and two cortical regions: the dorsolateral and medial prefrontal cortices.

**Conclusions:**

In a resting-state condition, Nx4 reduced the prefrontal cortex gFCD and strengthened the functional coupling between the amygdala and the prefrontal cortex that is relevant for emotion regulation and the stress response. Further studies should elaborate whether this mechanism represents enhanced regulatory control of the amygdala at rest and, consequently, to a diminished susceptibility to stress. ClinicalTrials.gov ID: NCT02602275.

**Impact statement:**

We provide global functional connectivity density maps as well as seed-based resting-state functional connectivity (rsFC) maps for the left centromedial amygdala from resting-state conditions in healthy but mild to moderately stressed participants. Our results elaborate on previous findings of neural effects in the amygdala by adding an rsFC dimension. We could then show an effect of the multicomponent drug Neurexan^®^ on functional connectivity in brain networks processing mood and anxiety, particularly in connections between the amygdala and the prefrontal cortex. This contributes to a growing mechanistic explanation for Neurexan's clinical stress-relieving effects.

## Introduction

### The resting-state brain as a basic functional organization

Brain activity is intrinsic, and it is present even in the absence of an externally prompted task in a so-called resting state. The resting-state brain can be understood as the basic functional organization of the brain. However, stress has an impact on the resting brain by changing the so-called resting-state functional connectivity (rsFC) in the regions associated with emotion regulation, namely between the amygdala and the cortical regions (Kim et al., 2011; van Marle et al., 2010; Veer et al., 2011).

### rsFC of the amygdala is affected by stress conditions

The amygdala is involved in emotional and motivational processing (Pessoa and Adolphs, 2010; Phelps and Anderson, 1997; Vuilleumier, 2005). It is considered to play a pivotal role in the detection of threatening cues and induction of the fear response (Phelps and LeDoux, 2005) as well as in initiating the stress response (van Marle et al., 2010; Veer et al., 2011).

The amygdala mediates the initial surge in vigilance, which prioritizes the information of sensory processing of potential stressors or threat-related stimuli (de Kloet et al., 2005; Quaedflieg et al., 2015). Several stress-related psychopathologies such as generalized anxiety disorder (Evans et al., 2008), post-traumatic stress disorder (Etkin and Wager, 2007; Rauch et al., 2000; Shin et al., 2005), or major depressive disorder (Hamilton et al., 2008) are associated with aberrant amygdala functions (Hölzel et al., 2010).

Through direct neuronal input from the thalamus, the amygdala rapidly reacts to stressors and orchestrates the stress response in the whole organism (Quaedflieg et al., 2015). Although afferent projections from many cortical areas are received by the basolateral amygdala, the centromedial amygdala (CeMA) sends the output to autonomic response-related areas involving the hypothalamus and the brain stem, and also to the anterior cingulate cortex (Vogt, 2018) that indirectly affects other cortical regions (Davis and Whalen, 2001).

The CeMA subregion was also found to be more strongly connected with the thalamus under threat conditions (Torrisi et al., 2018) and with the cerebellum, thalamus, and midbrain in patients suffering from generalized anxiety disorder (Etkin et al., 2004).

Functional magnetic resonance imaging (fMRI) has been used as a promising tool to understand the effects of stress on different brain regions and networks during stress tasks (de Kloet et al., 2005; Veer et al., 2011). Interestingly, stress induction before assessing rsFC influences the subsequent rsFC. More concretely, the brain at rest is affected by a preceding stress task. For the amygdala, the functional connectivity (FC) was shown to be perturbed during the experience of acute stress as well as thereafter at rest (Maron-Katz et al., 2016; Vaisvaser et al., 2013).

Even without any acute stress induction, patients with stress-related psychiatric conditions show altered rsFC: The amygdala rsFC was affected in patients with generalized anxiety disorder (Etkin et al., 2009), major depressive disorder (Cullen et al., 2014), post-traumatic stress disorder (Brown et al., 2014), and anxiety disorders (Hahn et al., 2011).

### Neurexan has demonstrated effects on the stress response

According to Lazarus and Folkman (1984), stress can be seen as a relationship between a person and the environment considered as taxing or exceeding the person's resources for coping. Prolonged stress exposure has a significant impact on the health status, potentially leading to a variety of stress-related symptoms such as nervousness, restlessness, insomnia, anxiety, depression, and hypertension (Slavich and Shields, 2018). A wide range of medications including benzodiazepines, serotonin reuptake inhibitors, and extracts of St John's-wort are used to overcome these symptoms, however, potentially demonstrating adverse side effects during long-term use (Apaydin et al., 2016; Bet et al., 2013).

Neurexan^®^ (Nx4; Heel GmbH, Baden-Baden, Germany), a plant-based medicinal drug composed of low concentrated herbal extracts of oat, coffee, passionflower, and a mineral salt, showed stress-relieving effects without adverse effects. In an observational study in human subjects with clinical symptoms of nervousness/restlessness, Nx4 led to a significant relief of the symptoms (Hubner et al., 2009).

In a randomized controlled trial in healthy subjects, Nx4 modulated the physiological stress response to an acute stress task, the Trier Social Stress Test, particularly by reducing salivary cortisol and plasma adrenaline (Doering et al., 2016). In a recently published analysis of the NEURIM trial, Nx4 reduced the susceptibility to distraction in an auditory discrimination task (Mayer et al., 2021) and reduced the amygdala activation in response to negative emotional stimuli compared with placebo (Herrmann et al., 2020) in mildly to moderately stressed healthy subjects.

### Objective of this publication: effects of Nx4 on rsFC before stress induction

In this publication, we focus on the effects of Nx4 on rsFC of the amygdala before stress induction. This sheds light on an altered functional connectivity at baseline on which acute stress induction is experienced and adds to the previously shown modification of the peripheral stress response and actual amygdala activations. The effects were assessed by using two different approaches: global functional connectivity density (gFCD) analysis and a seed-based approach.

The two analyses were defined as the second and third primary end-points of the NEURIM trial, respectively. As a basic network analysis, gFCD attempts to identify highly connected functional hubs (Tomasi and Volkow, 2010, 2011). It reveals how connected a voxel is but not the regions to which this voxel is connected (Lv et al., 2018). In contrast, the seed-voxel connectivity approach reveals the temporal similarity between a predefined seed region and each other region of the brain.

Based on the importance of rsFC of the left CeMA for the stress response, we hypothesized that in a resting-state condition before any stress induction, Nx4 leads to modifications of the left CeMA FC compared with placebo in terms of a reduction in amygdala gFCD (primary end-point 2) and a modification of the amygdala-centered rsFC (primary end-point 3).

## Materials and Methods

### Trial design

We analyzed resting-state fMRI (rsfMRI) data collected in the NEURIM trial (ClinicalTrials.gov identifier: NCT02602275; registered October 28, 2015) whose first primary end-point has been previously published (Herrmann et al., 2020). The study was approved by the Ethics Committee of the University of Magdeburg and the responsible authority (Federal Institute for Drugs and Medical Devices). It was conducted in accordance with the 1996 Declaration of Helsinki, the principles of Good Clinical Practice (GCP) and all applicable national laws and regulations. The study was conducted as a randomized, placebo-controlled, double-blind, two-treatment, two-period crossover trial with 1:1 randomization of the two treatment sequences, Nx4-Placebo and Placebo-Nx4.

Eligible participants were healthy males, aged 31 to 59 years, with mild to moderate chronic stress defined by a Trier Inventory for Chronic Stress—Screening Scale for Chronic Stress (TICS-SSCS) Score of ≥9 and ≤36 as well as a Perceived Stress Scale (PSS) of >9. A total of 40 participants were planned to be included at a single site at the Clinical Affective Neuroimaging Laboratory (CANLAB, Magdeburg, Germany).

**FIG. 1.**
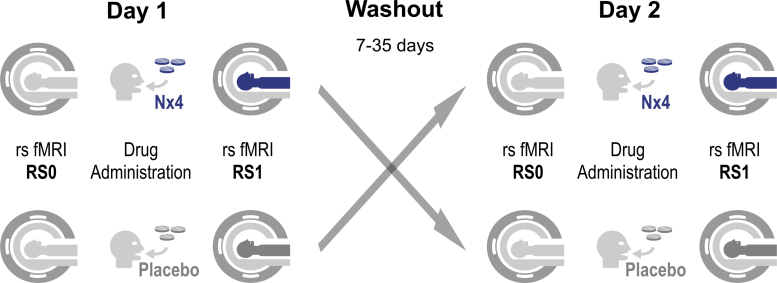
Study flow for primary end-points 2 and 3 evaluating rsFC without prior stress induction. On both study days, baseline rsfMRI scans were acquired immediately before dosing (RS0) and another rsfMRI about 1-h post-dose (RS1) without any stress-related tasks in between. Participants of the crossover trial received Nx4 on day 1 and placebo on day 2 or *vice versa*. A total of four rsfMRI scans per participant were performed for primary end-points 2 and 3. Nx4, Neurexan^®^; rsFC, resting-state functional connectivity; rsfMRI, resting-state functional magnetic resonance imaging.

Participants received a single dose (three tablets) of Nx4 or placebo on study day 1 and, after a 7 to 35 day washout period, the respective alternative treatment on study day 2. On both days, a variety of test procedures with several electroencephalography (EEG), EEG/fMRI, and psychological test sessions were performed as previously described (Herrmann et al., 2020). This publication describes the analysis of the rsfMRI data acquired immediately before dosing (RS0) and 40 to 60 min post-dose (RS1) before any stress induction (Fig. 1).

The justification of the chosen time points was based on a previous study in humans by Dimpfel (2019) that reported an effect of Nx4 on spectral EEG from 1 h after drug intake onward. To minimize any confounding effects of circadian rhythm, the rsfMRI measurements were performed at almost the same time of the day, in the afternoon.

### Primary end-points of the NEURIM trial described in this publication

Six primary end-points were defined in a hierarchical order beforehand. Multiplicity due to multiple primary hypotheses was controlled by means of the principle of *a priori* ordered hypotheses. The effect of Nx4 on primary end-point 1, a reduced left CeMA activation in response to negative emotional stimuli (emotional face matching in Harriri Task), has been previously published (Herrmann et al., 2020). In the current publication, we report on primary end-points 2 and 3. In contrast to primary end-point 1, primary end-points 2 and 3 evaluate FC in a resting-state condition before stress induction. The effect of Nx4 on rsFC of the left CeMA was assessed by gFCD (primary end-point 2) and a seed-based approach (primary end-point 3).

#### Primary end-point 2

Interaction of time and drug, driven by significantly greater reductions of resting-state amygdala gFCD in verum compared with placebo conditions.

#### Primary end-point 3

Interaction of time and drug, driven by significantly greater changes of resting-state amygdala-seeded connectivities in verum compared with placebo conditions.

### fMRI acquisition

A Philips 3T scanner was used for fMRI data acquisition. Structural T1-weighted images for spatial normalization were measured by using a turbo field echo sequence with the following parameters: 274 sagittal slices covering the whole brain, flip angle = 8°, 256 × 256 matrix, and voxel size 0.7 × 0.7 × 0.7 mm^3^. For the resting-state scans both before and after drug administration (RS0 and RS1), 355 volumes of T2*-weighted echo-planar images were acquired for each session with the following parameters: 34 axial slices covering the whole brain, repetition time = 2000 ms, echo time = 30 ms, flip angle = 90°, 96 × 94 matrix, field of view = 240 × 240 mm^2^, and voxel size = 2.5 × 2.5 × 3 mm^3^.

### fMRI preprocessing

Data from fMRI were preprocessed by using MATLAB 2018 (The Mathworks), SPM12 (Statistical parametric mapping software, SPM; Wellcome Department of Imaging Neuroscience, United Kingdom) and the CONN toolbox (Whitfield-Gabrieli and Nieto-Castanon, 2012). The first five volumes were discarded to allow the MR signal to achieve T1 equilibration. The images were first corrected for the acquisition time differences between slices and then realigned to the first volume to correct for head motion between volumes. The anatomical T1 images were coregistered to match the functional images and then segmented into gray matter and white matter. Coregistered rsfMRI data were normalized into the Montreal Neurological Institute space.

The physiological noise was reduced by (1) regressing out the first five principal components from the white matter and cerebrospinal fluid by using the component-based noise reduction method (CompCor) strategy and the 12-rigid body realignment parameters, and (2) removing a first-order polynomial trend before filtering the data to the infra-low frequency band (0.01–0.1 Hz). Importantly, global signal removal was not performed to avoid falsely increasing the anti-correlation between time series (Murphy et al., 2009). Instead, the CompCor has been proposed to sufficiently reduce the effect of physiological fluctuations on fMRI time series (Behzadi et al., 2007).

The mean image intensity and standard deviation were calculated to quantify micro-movement in resting-state measures by using the ART toolbox implemented in CONN. The intermediate threshold was used to detect and scrub bad volumes. A participant/condition was excluded if its volumes with large head motion exceeded 30% of the whole time-course or if any of the head motion parameters exceeded 3 mm. Functional images were re-sampled with a resolution of 3 × 3 × 3 mm^3^.

### gFCD as basic analysis to identify highly connected functional hubs

Highly connected functional hubs were identified by gFCD analysis. The number of voxels with a positive rsFC larger than the predefined threshold at a whole brain level was evaluated. Pearson correlation was used to assess the strength of rsFC, *C_ij_*, between voxels *i* and *j*. Consistent with prior gFCD studies (Tomasi and Volkow, 2010, 2011), a positive correlation threshold of *r* > 0.6 was used to compute the binary connectivity coefficients, *a_ij_* = 1 (if *C_ij_* > 0.6) or *a_ij_* = 0 (if *C_ij_* ≤ 0.6). A low threshold (e.g., *r* > 0.4) could lead to potential spurious weak correlations, whereas an excessive thresholding (e.g., *r* > 0.7) may result in lower sensitivities. The gFCD was calculated as the total number of edges for voxel *i*, that is, significant correlations (at *C_ij_* > 0.6) between voxel *i* and all voxels in the gray matter:


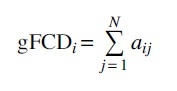


The gFCD value in each voxel (K) was divided by the global mean value (K_0_) to improve the normality of the data (K/K_0_). To assess the robustness of the chosen threshold, gFCD maps were also calculated for the thresholds of *r* > 0.4 and *r* > 0.5. The gFCD maps of each resting-state session from each participant were finally smoothed by using a Gaussian kernel of 8-mm full-width-half-maximum (FWHM) before statistical analysis was performed.

### Seed-based rsFC to identify connectivities between the left CeMA and each voxel in the whole brain

**FIG. 2.**
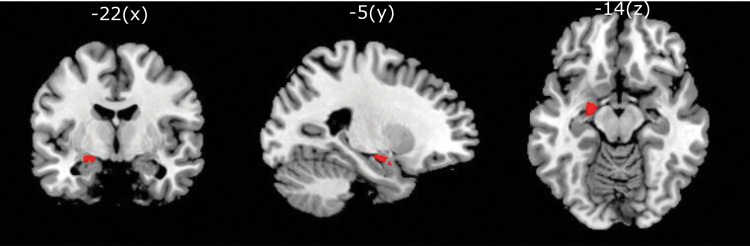
Definition of left CeMA (shown in red) for seed-based FC (MNI coordinates −22 − 5 −14) provided by the Anatomy Toolbox (Eickhoff et al., 2005). CeMA, centromedial amygdala; FC, functional connectivity; MNI, Montreal Neurological Institute.

Left CeMA was defined as the seed on the basis of probabilistic cytoarchitectonic maps (Amunts et al., 2005) provided by the Anatomy Toolbox (Eickhoff et al., 2005). These maps were thresholded at >80% probability for the CeMA (Fig. 2). The time course of the average pre-processed BOLD signal within the seed region was then correlated with the signals in each voxel in the whole brain. The Pearson correlation coefficients were transformed to *Z* values (Fisher's *Z*), resulting in a map representing the voxel-wise strength of rsFC to the seed region (Fisher's Z transformed functional connectivity [zFC] map). Finally, the zFC maps of the different time points were smoothed by using an 8-mm FWHM kernel before entering statistical analysis.

### Statistical analysis

The effect of Nx4 on resting-state metrics was defined as the second and third of the *a priori* ordered chain of primary end-points of the NEURIM clinical trial. Both end-points deal with the effect of Nx4 on the neural responses of the amygdala during resting state, with primary end-point 2 focusing on the gFCD changes and primary end-point 3 investigating the left CeMA seed-based rsFC changes. The analyses addressed the following questions.

#### Reproducing previous reports of gFCD and amygdala-seeded FC maps

To validate the resting-state metrics (gFCD map and CeMA seed-based FC map) across the whole brain, whole-brain one-sample *T*-tests were performed in SPM on RS0 data from day 1. Prominent functional hubs were indicated by gFCD values significantly greater than 1. Brain areas with positive or negative connectivity to the left CeMA seed were indicated by FC values significantly different from 0 (zero). Significance was assessed after correcting for multiple comparisons on peak level, using a family-wise error rate (FWE)-corrected *p* < 0.05 for gFCD map and a false discovery rate-corrected *p* < 0.05 for CeMA-seeded FC map.

#### Nx4 effect on gFCD of the amygdala (primary end-point 2)

To examine the effect of Nx4 on gFCD of the left CeMA, a within-subject repeated-measures ANOVA with main effects of time (RS0 vs. RS1) and drug (placebo vs. Nx4) and their interaction (time × drug) was performed in SPSS (IBM SPSS Statistics version 23). The age of the participants and the treatment sequence (Nx4-placebo or placebo-Nx4) were included as covariates. In addition, as an exploratory analysis, whole-brain gFCD was analyzed in SPM to investigate Nx4s effect on the gFCD of other brain regions.

#### Nx4 effect on seed-based rsFC of the amygdala (primary end-point 3)

To examine the effect of Nx4 on seed-based rsFC of the left CeMA, a whole-brain within-subject ANOVA with main effect of time (RS0 vs. RS1), main effect of drug (placebo vs. verum), and their interaction (time × drug) was performed in SPM. The age of the participants and the treatment sequence (Nx4-placebo or placebo-Nx4) were included as covariates. Significance was assessed after correcting for multiple comparisons by using cluster-wise FWE correction.

All voxels surpassing an initial height threshold of *p* < 0.001 entered a cluster with a threshold of cluster-level FWE-corrected *p* < 0.05. *Post hoc* analyses were done by using the mean FC values of the seed-based analysis. The mean FC values between left CeMA and the obtained significant clusters were extracted and compared between placebo and Nx4 at each resting state (RS0 and RS1).

## Results

### Number of participants, baseline characteristics, and safety

The study showed no indication for a safety risk after a single dose treatment with 3 tablets of Nx4, as none of the 39 drug administered participants suffered an adverse event in the observation period, neither under Nx4 nor under placebo.

A total number of 40 participants were included. All participants were healthy males, 31 to 59 years old and had mild to moderate levels of stress. Twenty participants were randomly assigned to each of the two treatment sequences, placebo first or Nx4 first. One participant was withdrawn due to an incidental baseline MRI finding before first drug administration. The remaining 39 participants received placebo or Nx4 and underwent rsfMRI scans on days 1 and 2. Data from one participant of the placebo first sequence were removed due to corrupted fMRI data at RS0 on day 1.

**FIG. 3.**
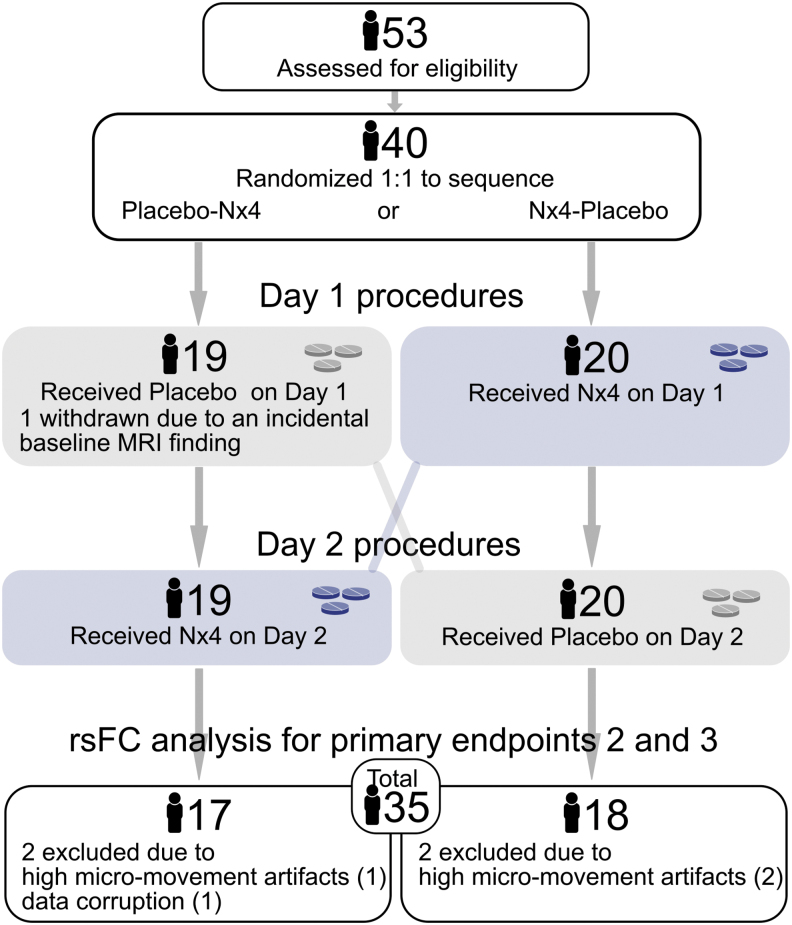
Number of participants in the two sequences of the cross-over trial, placebo first and Nx4 first, analyzed for primary end-points 2 and 3 evaluating rsFC without prior stress induction.

Data from three participants (one from placebo first sequence, two from Nx4 first sequence) did not meet the quality requirements due to high micro-movement artifacts in at least one of the four sessions (placebo_RS0, placebo_RS1, Nx4_RS0, Nx4_RS1). Therefore, out of the 39 participants finishing the study, 35 participants (18 participants receiving Nx4 first and 17 participants receiving placebo first) were included in this statistical analysis (Fig. 3). There were no substantial differences between the two sequences in terms of age and stress scores, as shown in Table 1.

**Table 1.** Mean Age and Baseline Perceived Stress Scores (±Standard Deviation) of the Two Study Sequences Receiving Nx4 First or Placebo First, Respectively

	Placebo-Nx4	Nx4-Placebo
*n*	17	18
Age	45.0 ± 9.9	42.8 ± 10.5
TICS-SSCS	15.4 ± 4.1	14.2 ± 3.7
PSS	15.5 ± 6.1	16.2 ± 5.7

Study sequences were balanced regarding the number of participants, age, and the two applied global measures of perceived stress; the TICS-SSCS as well as a the PSS. Participants showed mild to medium perceived stress scores (TICS-SSCS Score of ≥9 and ≤36 as well as a PSS of >9).

Nx4, Neurexan^®^; PSS, Perceived Stress Scale; TICS-SSCS, Trier Inventory for Chronic Stress—Screening Scale for Chronic Stress.

### Primary end-point 2: effect of Nx4 on gFCD of the amygdala was not significant

**FIG. 4.**
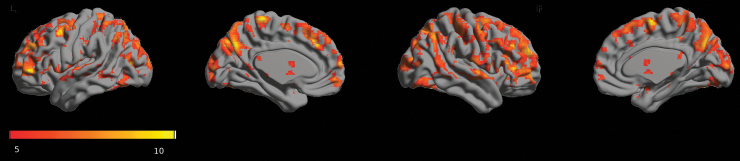
Statistical parametric mapping of prominent gFCD hubs (voxel-wise FWE-corrected *p* < 0.05) using baseline resting-state data (RS0) at day 1. Orange-yellow colorbar indicates the *T* values. Highly connected functional hubs were identified mainly in the cortex. FWE, family-wise error rate; gFCD, global functional connectivity density.

For baseline RS0 data from day 1, we identified prominent gFCD-hubs in the visual cortex and posterior ventral-medial parietal cortex. Insula, auditory, dorsal parietal, and ventral frontal cortex also showed intense gFCD-hubs, whereas the availability of gFCD-hubs in the subcortical areas was minimal (Fig. 4). In the amygdala, we did not find a prominent hub. The whole-brain gFCD maps were in line with previously reported findings (Tomasi and Volkow, 2010, 2011).

As primary end-point 2 of the NEURIM study, we investigated the effect of Nx4 on gFCD of the left CeMA. The effect of Nx4 versus placebo on gFCD of the left CeMA did not reach a level of significance. Two-way repeated-measures ANOVA (with main factors of treatment and time) showed no significant effect of the drug (all *p* values >0.9; for effect size, all partial η^2^ values <0.00), and no significant drug × time interaction (all *p* values >0.35; for effect size, all partial η^2^ values <0.025).

Since we controlled for multiplicity due to multiple primary hypotheses by means of the principle of *a priori* ordered hypotheses and primary end-point 2 was not met, all subsequent analyses were regarded as purely exploratory.

### Nx4 significantly reduced gFCD of the medial prefrontal cortex

**FIG. 5.**
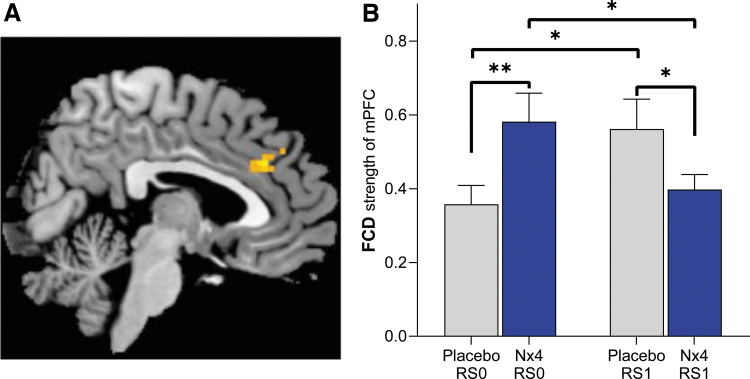
**(A)** Whole-brain analysis of FCD showed a significant interaction between drug and time for FCD in the mPFC. Results were reported at uncorrected *p* < 0.005, cluster size >10. **(B)** Strength of FCD in the mPFC at baseline (RS0) and 1-h post-dose (RS1) under Nx4 or placebo. Data are given as mean with standard error of mean. Significant differences are indicated as **p < 0.01 and *p < 0.05. mPFC, medial prefrontal cortex.

Using an exploratory approach, we analyzed the effect of Nx4 versus placebo on whole-brain gFCD by means of a two-way repeated-measures ANOVA. A significant effect of Nx4 was found for gFCD of the medial prefrontal cortex (mPFC; Fig. 5A; drug × time interaction with uncorrected *p* < 0.005, cluster-size >10). *Post hoc T*-tests revealed that gFCD strength of the mPFC was significantly lower after Nx4 treatment compared with placebo at RS1 [Fig. 5B; *t*(34) = −2.04, *p* = 0.048]. For the Nx4 condition, gFCD of the mPFC decreased significantly from baseline (RS0) to the post-dose resting-state measurement [RS1; *t*(34) = −2.18, *p* = 0.036]. For the placebo condition, a significant increase from RS0 to RS1 was observed [*t*(34) = 2.62, *p* = 0.012].

### Primary end-point 3: Nx4 significantly modulated seed-based rsFC between the left CeMA and PFC

**FIG. 6.**
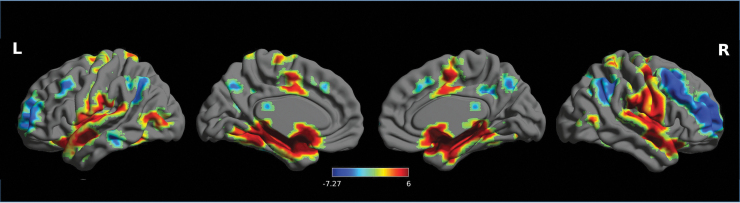
Whole-brain functional connectivity map from left CeMA using baseline resting-state data (RS0) at day 1. All the results were reported at *p* < 0.05, corrected for multiple comparisons using FDR correction. Colorbar indicates *T* values. FDR, false discovery rate.

Using baseline resting-state data (RS0) at day 1, rsFC of the left CeMA (our prespecified region of interest) was analyzed by whole-brain one-sample *T*-tests. The left CeMA showed significant positive rsFC with the mid-cingulate cortex, amygdala, insula, thalamus, putamen, pallidum, and brainstem, and negative rsFC with the precuneus, middle frontal gyrus, and occipital gyrus (Fig. 6). The proposed whole-brain rsFC maps from the left CeMA were in line with previous findings (Gorka et al., 2018; Roy et al., 2009).

**FIG. 7.**
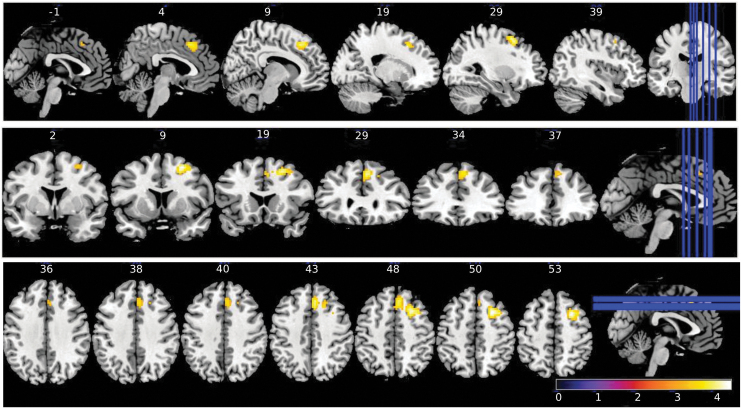
Brain regions showing significant (cluster-wise FWE-corrected at *p* < 0.05) drug × time interaction on seed-based rsFC for left CeMA. Significant effects of Nx4 versus placebo were observed for the rsFC between left CeMA and two cortical regions, the dlPFC and the mPFC. Colorbar indicates *T* values. dlPFC, dorsolateral prefrontal cortex.

For primary end-point 3 of the NEURIM study, we investigated the effect of Nx4 on rsFC data with the left CeMA as the prespecified seed. Significant effects of Nx4 versus placebo were observed for the rsFC between the left CeMA and two cortical regions: the dorsolateral prefrontal cortex (dlPFC) and the mPFC: Whole-brain within-subject ANOVA with main factors of time (RS0 vs. RS1) and drug (placebo vs. Nx4) showed a significant drug × time interaction for both, dlPFC and mPFC (Fig. 7 and Table 2). Since primary end-point 2 of the *a priori* ordered chain of primary end-points was not met, the results of primary end-point 3 were regarded as exploratory.

**Table 2.** Brain Regions Showing Significant (Cluster-Wise Family-Wise Error Rate Corrected *p* < 0.05) Drug × Time Interaction on Resting-State Functional Connectivity with Left Centromedial Amygdala

Contrast	Cluster level *p*FWE	k	T	MNI (*x y z*), mm	Label
Placebo>Nx4	0.008	139	4.47	27 9 54	dlPFC
	0.045	89	4.28	6 27 45	mPFC

dlPFC, dorsolateral prefrontal cortex; MNI, Montreal Neurological Institute; mPFC, medial prefrontal cortex; pFWE, p family-wise error.

**FIG. 8.**
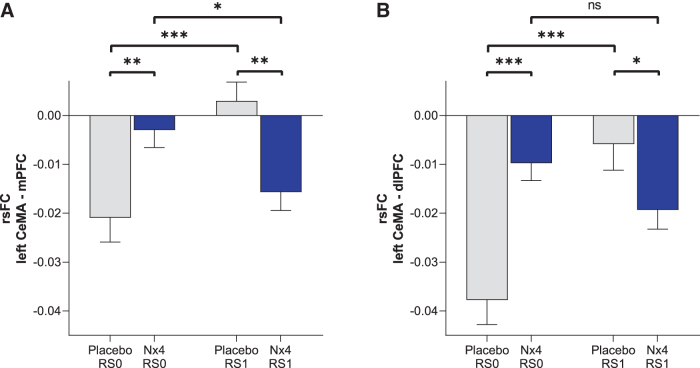
Strength of rsFC between left CeMA and **(A)** mPFC and **(B)** dlPFC at baseline (RS0) and 1 h post-dose (RS1) under Nx4 or placebo. Data are given as mean with standard error of mean. Significant differences are indicated as ***p < 0.001, **p < 0.01, and *p < 0.05; ns, not significant.

Negative rsFC between the left CeMA and two cortical regions, dlPFC and mPFC, were observed. The strengths of these negative rsFC were significantly greater (more negative) after Nx4 treatment compared with placebo (Fig. 8): *Post hoc T*-tests revealed significant differences between Nx4 and placebo for the left CeMA-mPFC FC [*t*(34) = −2.17, *p* = 0.036] and left CeMA-dlPFC FC [*t*(34) = −3.55, *p* = 0.001] at the post-dose resting state (RS1). Nx4, therefore, led to a strengthened functional coupling between the left CeMA and the dlPFC as well as the mPFC.

Looking at the time course from RS0 to RS1, we observed that for the Nx4 condition, rsFC between left CeMA and PFC became more negative from baseline (RS0) to the post-dose measurement (RS1). The difference between RS0 and RS1 was significant for the mPFC [*t*(34) = −2.28, *p* = 0.028] and not significant for the dlPFC [*t*(34) = −1.89, *p* = 0.06]. In contrast, for the placebo condition, the negative rsFC between the left CeMA and PFC became less prominent from RS0 to RS1 (dlPFC [*t*(34) = −5.03, *p* < 0.001]; mPFC [*t*(34) = −4.08, *p* < 0.001]).

### Nx4 showed an effect on rsFC within 1-h post-dose

The effect of Nx4 versus placebo on rsFC was observed during the rsfMRI session conducted 40 to 60 min after a single oral dose of Nx4. Within 1-h post-dose, Nx4 led to significantly reduced gFCD strength of the mPFC as well as to significantly strengthened negative coupling of left CeMA and dlPFC as well as mPFC.

## Discussion

### No prominent gFCD-hubs in the amygdala and no effect of Nx4 on the amygdala gFCD

In this publication, we explored the effect of Nx4 on amygdala-centered rsFC. We started our analyses with a gFCD approach for the amygdala. As a data-driven method, gFCD is broadly applied to identify the major cortical and subcortical FC hubs at rest. We, nevertheless, aimed at including the amygdala for gFCD analysis due to its key anatomical and functional importance for cortical and subcortical structures. However, no prominent hubs were identified in the amygdala.

This finding might be explained by the fact that no stress task was involved from RS0 to RS1 and the participants suffered only from mild to moderate stress. Consequently, we were not able to detect a significant effect of Nx4 on gFCD of the left CeMA either. The lacking effect on primary outcome 2 led us to stop the original statistical path of sequential hypothesis. All subsequent findings were, therefore, regarded as exploratory findings.

### Nx4 reduced gFCD of the mPFC

Using an exploratory approach, we then applied whole-brain gFCD without a regional hypothesis. The analysis identified the mPFC as a relevant functional hub affected by Nx4. The mPFC is known to be involved in regulatory control of behavior during the experience of an aversive event such as stress or fear. For the mPFC, the gFCD strength was significantly lower after Nx4 treatment compared with placebo, suggesting a relaxing effect of Nx4.

### Nx4 modulated seed-based rsFC of the left CeMA and PFC

The seed-based approach supported the modulatory effect of Nx4 on gFCD of the PFC. We demonstrated that Nx4 significantly enhanced the negative rsFC between the CeMA and mPFC as well as dlPFC. We found that left CeMA activity at rest was negatively correlated with the dlPFC and mPFC activity and the strength of this resting-state CeMA-mPFC and CeMA-dlPFC functional connectivity is enhanced significantly after Nx4 as compared with placebo.

### Nx4 effect on rsFC between the CeMA and PFC can be related to mood and anxiety processing

The pathways between the amygdala and PFC play a crucial role in the integration of affective information into cognitive processing (Etkin et al., 2011; Gold et al., 2016; Monk et al., 2008). According to the cognitive control model of emotion regulation, the cross-talk between the amygdala and PFC (both dlPFC and mPFC) and the anterior cingulate cortex represents the neural circuit of emotion regulation (Ochsner and Gross, 2005). The dlPFC, in particular, plays an important role in the disengagement from negative stimuli and thoughts, and it exerts a suppressing effect on the amygdala (D'Esposito et al., 1999; Ochsner and Gross, 2005).

In fact, the strength of the cross-talk between the mPFC and the amygdala at rest is proposed to underlie the emotional regulation capacity (Kim et al., 2011). Under stressful conditions or in patients with anxiety disorders, rsFC between the amygdala and mPFC is altered (Kim et al., 2011). Both increased medial and lateral PFC activity was shown to accompany decreased amygdala activity during emotional regulation tasks (Ochsner and Gross, 2005; Simmons et al., 2008), which is also linked to everyday application of emotional regulation strategies (Drabant et al., 2009).

Previous studies showed that this negative FC between amygdala and mPFC is correlated with a lower level of anxiety (Gee et al., 2013; Kim et al., 2011, 2012; Vytal et al., 2014). Moreover, it was shown that increased emotional regulation capacity is accompanied by a developmental shift from positive to negative FC in the human amygdala–PFC circuitry, indicating enhanced top-down improvements in emotion regulation in later stages of life (Gee et al., 2013).

In line with these findings and considering the widely acknowledged relationship between emotion regulation and top-down modulatory effect of the dlPFC and mPFC on the amygdala (Hare et al., 2008; Hariri et al., 2003; Kim et al., 2011; Pezawas et al., 2005), the enhanced negative amygdala-PFC rsFC under Nx4 in this study suggests that Nx4 strengthens the functional coupling between the amygdala and the prefrontal brain regions relevant to processing mood and anxiety. This effect may contribute to the stress remediating activity of Nx4.

### Limitations

We have observed differences between the placebo and Nx4 condition for gFCD in the mPFC and rsFC between the left CeMA and mPFC as well as dlPFC at baseline (RS0) that are interpreted as random differences. Our hypothesis was, however, based on changes of rsFC from baseline to post-drug ingestion (time × drug interaction) that can be regarded as independent from the actual baseline levels.

Our participants were all males and chosen based on the PSS and TICS-SSCS stress scores to ensure they were, in principle, susceptible to stress, but not chronically stressed to avoid a ceiling effect of stress sensitivity. The exclusion of female participants and participants with extreme stress scores in either direction limits the generalizability. A study in participants of both genders with greater everyday burden or in patients with stress-induced diseases would be recommended for follow-up. For a meaningful conclusion on the clinical relevance, further studies in clinically well-defined patient groups would be required that assess clinically relevant outcome measures in parallel to fMRI.

## Conclusion

In a resting-state condition in healthy, mildly to moderately stressed men, Nx4 reduced the gFCD of the PFC and strengthened the functional coupling between left CeMA and PFC that is relevant for emotional regulation and the stress response. Further studies should elaborate whether this mechanism represents enhanced regulatory control of the amygdala at rest and consequently to a diminished susceptibility to stress.

## Data Availability

The datasets presented in this article are not readily available, because data belong to the sponsor of the clinical trial (Heel GmbH) and require previous consent of the sponsor. Requests to access the datasets should be directed to martin.walter@med.uni-jena.de. The MATLAB code used in the article is available at https://github.com/tarachandtaak/MS2_Nx4.git

## References

[B1] Amunts K, Kedo O, Kindler M, et al. 2005. Cytoarchitectonic mapping of the human amygdala, hippocampal region and entorhinal cortex: intersubject variability and probability maps. Anat Embryol 210:343–352. 10.1007/s00429-005-0025-5 16208455

[B2] Apaydin EA, Maher AR, Shanman et al. 2016. A systematic review of St. John's wort for major depressive disorder. Syst Rev 5:148. 10.1186/s13643-016-0325-2 PMC5010734 27589952

[B3] Behzadi Y, Restom K, Liau J, et al. 2007. A component based noise correction method (CompCor) for BOLD and perfusion based fMRI. Neuroimage 37:90–101. 17560126 10.1016/j.neuroimage.2007.04.042 PMC2214855

[B4] Bet PM, Hugtenburg JG, Penninx BWJH, et al. 2013. Side effects of antidepressants during long-term use in a naturalistic setting. Eur Neuropsychopharmacol 23:1443–1451. 23726508 10.1016/j.euroneuro.2013.05.001

[B5] Brown VM, LaBar KS, Haswell CC, et al. 2014. Altered resting-state functional connectivity of basolateral and centromedial amygdala complexes in posttraumatic stress disorder. Neuropsychopharmacology 39:351–359. 23929546 10.1038/npp.2013.197 PMC3870774

[B6] Cullen KR, Westlund MK, Klimes-Dougan B, et al. 2014. Abnormal amygdala resting-state functional connectivity in adolescent depression. JAMA Psychiatry 71:1138–1147. 25133665 10.1001/jamapsychiatry.2014.1087 PMC4378862

[B7] Davis M, Whalen PJ. 2001. The amygdala: vigilance and emotion. Mol Psychiatry 6:13–34. 11244481 10.1038/sj.mp.4000812

[B8] de Kloet ER, Joëls M, Holsboer F. 2005. Stress and the brain: from adaptation to disease. Nat Rev Neurosci 6:463–475. 15891777 10.1038/nrn1683

[B9] D'Esposito M, Postle BR, Jonides J, et al. 1999. The neural substrate and temporal dynamics of interference effects in working memory as revealed by event-related functional MRI. Proc Natl Acad Sci U S A 96:7514–7519. 10377446 10.1073/pnas.96.13.7514 PMC22117

[B10] Dimpfel W. 2019. Effects of Neurexan on stress-induced changes of spectral EEG power: a double-blind, randomized, placebo-controlled, crossover exploratory trial in human volunteers. World J Neurosci 9:100–112.

[B11] Doering BK, Wegner A, Hadamitzky M, et al. 2016. Effects of Neurexan ® in an experimental acute stress setting—an explorative double-blind study in healthy volunteers. Life Sci 146:139–147. 26772822 10.1016/j.lfs.2015.12.058

[B12] Drabant EM, McRae K, Manuck SB, et al. 2009. Individual differences in typical reappraisal use predict amygdala and prefrontal responses. Biol Psychiatry 65:367–373. 18930182 10.1016/j.biopsych.2008.09.007 PMC2855682

[B13] Eickhoff SB, Stephan KE, Mohlberg H, et al. 2005. A new SPM toolbox for combining probabilistic cytoarchitectonic maps and functional imaging data. Neuroimage 25:1325–1335. 15850749 10.1016/j.neuroimage.2004.12.034

[B14] Etkin A, Egner T, Kalisch R. 2011. Emotional processing in anterior cingulate and medial prefrontal cortex. Trends Cogn Sci 15:85–93. 21167765 10.1016/j.tics.2010.11.004 PMC3035157

[B15] Etkin A, Klemenhagen KC, Dudman JT, et al. 2004. Individual differences in trait anxiety predict the response of the basolateral amygdala to unconsciously processed fearful faces. Neuron 44:1043–1055. 15603746 10.1016/j.neuron.2004.12.006

[B16] Etkin A, Prater KE, Schatzberg, AF, et al. 2009. Disrupted amygdalar subregion functional connectivity and evidence of a compensatory network in generalized anxiety disorder. Arch Gen Psychiatry 66:1361–1372. 19996041 10.1001/archgenpsychiatry.2009.104 PMC12553334

[B17] Etkin A, Wager TD. 2007. Functional neuroimaging of anxiety: a meta-analysis of emotional processing in PTSD, social anxiety disorder, and specific phobia. Am J Psychiatry 164:1476–1488. 17898336 10.1176/appi.ajp.2007.07030504 PMC3318959

[B18] Evans S, Ferrando S, Findler M, et al. 2008. Mindfulness-based cognitive therapy for generalized anxiety disorder. J Anxiety Disord 22:716–721. 17765453 10.1016/j.janxdis.2007.07.005

[B19] Gee DG, Humphreys KL, Flannery J, et al. 2013. A developmental shift from positive to negative connectivity in human amygdala-prefrontal circuitry. J Neurosci 33:4584–4593. 23467374 10.1523/JNEUROSCI.3446-12.2013 PMC3670947

[B20] Gold AL, Shechner T, Farber MJ, et al. 2016. Amygdala-cortical connectivity: associations with anxiety, development, and threat. Depress Anxiety 33:917–926. 27699940 10.1002/da.22470 PMC5096647

[B21] Gorka AX, Torrisi S, Shackman AJ, et al. 2018. Intrinsic functional connectivity of the central nucleus of the amygdala and bed nucleus of the stria terminalis. Neuroimage 168:392–402. 28392491 10.1016/j.neuroimage.2017.03.007 PMC5630489

[B22] Hahn A, Stein P, Windischberger C, et al. 2011. Reduced resting-state functional connectivity between amygdala and orbitofrontal cortex in social anxiety disorder. Neuroimage 56:881–889. 21356318 10.1016/j.neuroimage.2011.02.064

[B23] Hamilton JP, Siemer M, Gotlib IH. 2008. Amygdala volume in major depressive disorder: a meta-analysis of magnetic resonance imaging studies. Mol Psychiatry 13:993–1000. 18504424 10.1038/mp.2008.57 PMC2739676

[B24] Hare TA, Tottenham N, Galvan A, et al. 2008. Biological substrates of emotional reactivity and regulation in adolescence during an emotional go-nogo task. Biol Psychiatry 63:927–934. 18452757 10.1016/j.biopsych.2008.03.015015 PMC2664095

[B25] Hariri AR, Mattay VS, Tessitore A, et al. 2003. Neocortical modulation of the amygdala response to fearful stimuli. Biol Psychiatry 53:494–501. 12644354 10.1016/s0006-3223(02)01786-9

[B26] Herrmann L, Vicheva P, Kasties V, et al. 2020. FMRI revealed reduced amygdala activation after nx4 in mildly to moderately stressed healthy volunteers in a randomized, placebo-controlled, cross-over trial. Sci Rep 10:3802. 32123197 10.1038/s41598-020-60392-w PMC7052227

[B27] Hölzel BK, Carmody J, Evans KC, et al. 2010. Stress reduction correlates with structural changes in the amygdala. Soc Cogn Affect Neurosci 5:11–17. 19776221 10.1093/scan/nsp034 PMC2840837

[B28] Hubner R, van Haselen R, Klein P. 2009. Effectiveness of the homeopathic preparation Neurexan^®^ compared with that of commonly used valerian-based preparations for the treatment of nervousness/restlessness—an observational study. ScientificWorldJournal 9:733–745. 19705035 10.1100/tsw.2009.95 PMC5823074

[B29] Kim JE, Dager SR, Lyoo IK. 2012. The role of the amygdala in the pathophysiology of panic disorder: evidence from neuroimaging studies. Biol Mood Anxiety Disord 2:20. 23168129 10.1186/2045-5380-2-20 PMC3598964

[B30] Kim M, Loucks R, Palmer A, et al. 2011. The structural and functional connectivity of the amygdala: from normal emotion to pathological anxiety. Behav Brain Res 223:403–410. 21536077 10.1016/j.bbr.2011.04.025 PMC3119771

[B31] Lazarus RS, Folkman S. 1984. Stress, Appraisal, and Coping. New York, NY: Springer Pub.

[B32] Lv H, Wang Z, Tong E, et al. 2018. Resting-state functional MRI: everything that nonexperts have always wanted to know. AJNR Am J Neuroradiol 39:1390–1399. 29348136 10.3174/ajnr.A5527 PMC6051935

[B33] Maron-Katz A, Vaisvaser S, Lin T, et al. 2016. A large-scale perspective on stress-induced alterations in resting-state networks. Sci Rep 6:21503. 26898227 10.1038/srep21503 PMC4761902

[B34] Mayer K, Krylova M, Alizadeh S, et al. 2021. Nx4 reduced susceptibility to distraction in an attention modulation task. Front Psychiatry 12:746215. 34912250 10.3389/fpsyt.2021.746215 PMC8667722

[B35] Monk CS, Telzer EH, Mogg K, et al. 2008. Amygdala and ventrolateral prefrontal cortex activation to masked angry faces in children and adolescents with generalized anxiety disorder. Arch Gen Psychiatry 65:568–576. 18458208 10.1001/archpsyc.65.5.568 PMC2443697

[B36] Murphy K, Birn RM, Handwerker DA, et al. 2009. The impact of global signal regression on resting state correlations: are anti-correlated networks introduced? Neuroimage 44:893–905. 18976716 10.1016/j.neuroimage.2008.09.036 PMC2750906

[B37] Ochsner KN, Gross JJ. 2005. The cognitive control of emotion. Trends Cogn Sci 9:242–249. 15866151 10.1016/j.tics.2005.03.010

[B38] Pessoa L, Adolphs R. 2010. Emotion processing and the amygdala: from a “low road” to “many roads” of evaluating biological significance. Nat Rev Neurosci 11:773–783. 20959860 10.1038/nrn2920 PMC3025529

[B39] Pezawas L, Meyer-Lindenberg A, Drabant EM, et al. 2005. 5-HTTLPR polymorphism impacts human cingulate-amygdala interactions: a genetic susceptibility mechanism for depression. Nat Neurosci 8:828–834. 15880108 10.1038/nn1463

[B40] Phelps EA, Anderson AK. 1997. Emotional memory: what does the amygdala do? Curr Biol 7:R311–R314. 9115384 10.1016/s0960-9822(06)00146-1

[B41] Phelps EA, LeDoux JE. 2005. Contributions of the amygdala to emotion processing: from animal models to human behavior. Neuron 48:175–187. 16242399 10.1016/j.neuron.2005.09.025

[B42] Quaedflieg CWEM, van de Ven V, Meyer T, et al. 2015. Temporal dynamics of stress-induced alternations of intrinsic amygdala connectivity and neuroendocrine levels. PLoS One 10:e0124141. 25946334 10.1371/journal.pone.0124141 PMC4422669

[B43] Rauch SL, Whalen PJ, Shin LM, et al. 2000. Exaggerated amygdala response to masked facial stimuli in posttraumatic stress disorder: a functional MRI study. Biol Psychiatry 47:769–776. 10812035 10.1016/s0006-3223(00)00828-3

[B44] Roy AK, Shehzad Z, Margulies DS, et al. 2009. Functional connectivity of the human amygdala using resting state fMRI. Neuroimage 45:614–626. 19110061 10.1016/j.neuroimage.2008.11.030 PMC2735022

[B45] Shin LM, Wright CI, Cannistraro PA, et al. 2005. A functional magnetic resonance imaging study of amygdala and medial prefrontal cortex responses to overtly presented fearful faces in posttraumatic stress disorder. Arch Gen Psychiatry 62:273–281. 15753240 10.1001/archpsyc.62.3.273

[B46] Simmons AN, Paulus MP, Thorp SR, et al. 2008. Functional activation and neural networks in women with posttraumatic stress disorder related to intimate partner violence. Biol Psychiatry 64:681–690. 18639236 10.1016/j.biopsych.2008.05.027 PMC2634744

[B47] Slavich GM, Shields GS. 2018. Assessing lifetime stress exposure using the stress and adversity inventory for adults (Adult STRAIN): an overview and initial validation. Psychosom Med 80:17–27. 29016550 10.1097/PSY.0000000000000534 PMC5757659

[B48] Tomasi D, Volkow ND. 2010. Functional connectivity density mapping. Proc Natl Acad Sci U S A 107:9885–9890. 20457896 10.1073/pnas.1001414107 PMC2906909

[B49] Tomasi D, Volkow ND. 2011. Association between functional connectivity hubs and brain networks. Cereb Cortex 21:2003–2013. 21282318 10.1093/cercor/bhq268 PMC3165965

[B50] Torrisi S, Gorka AX, Gonzalez-Castillo J, et al. 2018. Extended amygdala connectivity changes during sustained shock anticipation. Transl Psychiatry 8:33. 29382815 10.1038/s41398-017-0074-6 PMC5802685

[B51] Vaisvaser S, Lin T, Admon R, et al. 2013. Neural traces of stress: cortisol related sustained enhancement of amygdala-hippocampal functional connectivity. Front Hum Neurosci 7:313. 23847492 10.3389/fnhum.2013.00313 PMC3701866

[B52] van Marle HJF, Hermans EJ, Qin S, et al. 2010. Enhanced resting-state connectivity of amygdala in the immediate aftermath of acute psychological stress. Neuroimage 53:348–354. 20621656 10.1016/j.neuroimage.2010.05.070

[B53] Veer IM, Oei NYL, Spinhoven P, et al. 2011. Beyond acute social stress: increased functional connectivity between amygdala and cortical midline structures. Neuroimage 57:1534–1541. 21664280 10.1016/j.neuroimage.2011.05.074

[B54] Vogt BA. 2018. Anxiety and fear from the perspective of cingulate cortex. J Depress Anxiety Forecast 1:1003.

[B55] Vuilleumier P. 2005. How brains beware: neural mechanisms of emotional attention. Trends Cogn Sci 9:585–594. 16289871 10.1016/j.tics.2005.10.011

[B56] Vytal KE, Overstreet C, Charney DR, et al. 2014. Sustained anxiety increases amygdala-dorsomedial prefrontal coupling: a mechanism for maintaining an anxious state in healthy adults. J Psychiatry Neurosci 39:321–329. 24886788 10.1503/jpn.130145 PMC4160361

[B57] Whitfield-Gabrieli S, Nieto-Castanon A. 2012. Conn: a functional connectivity toolbox for correlated and anticorrelated brain networks. Brain Connect 2:125–141. 22642651 10.1089/brain.2012.0073

